# Oral administration of kynurenic acid delays the onset of type 2 diabetes in Goto-Kakizaki rats

**DOI:** 10.1016/j.heliyon.2023.e17733

**Published:** 2023-06-27

**Authors:** Delong Zhen, Lina Ding, Bao Wang, Xiaolei Wang, Yanli Hou, Wenyu Ding, Bernard Portha, Junjun Liu

**Affiliations:** a Shandong Institute of Endocrine and Metabolic Diseases, Shandong First Medical University & Shandong Academy of Medical Sciences, Jinan, China; b Laboratoire B2PE (Biologie et Pathologie du Pancréas Endocrine), Unité BFA (Biologie Fonctionnelle et Adaptive), CNRS UMR 8251, Université Paris-Cité, Paris, France

**Keywords:** KYNA, Energy metabolism, Liver, UCPs, Diabetes

## Abstract

Kynurenic acid (KYNA) is an endogenous catabolite of tryptophan that has been found to demonstrate neuroprotective properties in psychiatric disorders. Recently, accumulating data have suggested that KYNA may also play a significant role in various metabolic diseases by stimulating energy metabolism in adipose tissue and muscle. However, whether KYNA can serves as an anti-diabetes agent has yet to be studied. In this study, we investigated the potential anti-diabetic effects of administering KYNA orally through drinking water in pre-diabetic Goto-Kakizaki rats and examined how this treatment may influence energy metabolism regulation within the liver. We found that hyperglycemic Goto-Kakizaki rats showed lower plasmatic KYNA levels compared to normal rats. Oral administration of KYNA significantly delayed the onset of diabetes in Goto-Kakizaki rats compared to untreated animals. Moreover, we found that KYNA treatment significantly increased respiration exchange ratio and promoted the energy expenditure by stimulating the expression of uncoupling protein (UCP). We confirmed that KYNA stimulated the UCP expression in HepG2 cells and mouse hepatocytes at mRNA and protein levels. Our study reveals that KYNA could potentially act as an anti-diabetic agent and KYNA-induced UCP upregulation is closely associated with the regulation of energy metabolism. These results provide further evidence for the therapeutic potential of KYNA in diabetes.

## Graphical abstract

**Figure:**
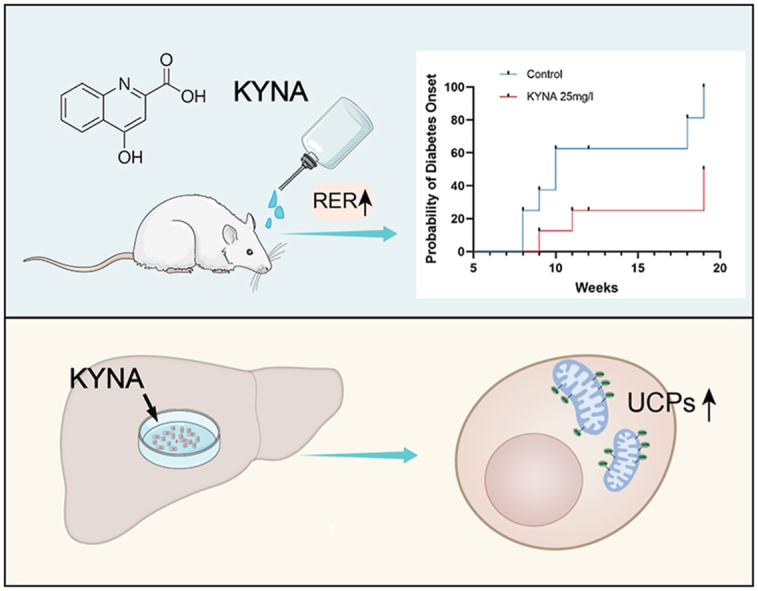

## Abbreviations

GK: Goto-Kakizaki rat
W: Wistar rat
STZ: Streptozotocin
HFD: High fat diet
TKP: Tryptophan/Kynurenine Pathway
Trp: Tryptophan
KYN: Kynurenine
3-HK: 3-hydroxykynurenine
5-HT: 5-hydroxytryptamine
KYNA: Kynurenic acid
QUIN: Quinolinic acid
RER: Respiration exchange ratio
UCP: Uncoupling protein

## 1 Introduction

Dietary tryptophan (Trp), apart from its structural role, is a primary source for various biologically active molecules, including serotonin, kynurenines, indoles and nicotinamide adenine dinucleotide (NAD^+^). Only minor quantities of Trp are used for the synthesis of proteins (1%) and serotonin (4%), whereas the remaining (95%) is converted along the Trp/kynurenine pathway (TKP), which yields a number of intermediates termed collectively kynurenines [[1], [2], [3], [4]]. Kynurenines include several metabolites displaying a wide range of biological actions that are often contrasting, such as cytotoxic/cytoprotective, oxidant/, antioxidant or pro-/anti-inflammatory. The canonical classification of kynurenines presents them as either protective, such as kynurenic acid (KYNA) or toxic, such as quinolinic acid or 3-hydroxykynurenine.

The role of kynurenines in the brain has been widely explored, with much of the research focused on the development of neurodegenerative disorders such as Huntington’s and Alzheimer’s diseases, as well as on psychiatric disorders such as depression. This is because Kynurenic acid (KYNA) is the sole endogenous antagonist of the N-methyl-d-aspartate receptor, which can help protect neurons from “excitotoxicity” [5]. More recent evidence suggests that alteration of kynurenine metabolism may also impact the pathogenesis of metabolic disorders such as diabetes mellitus [6,7], and cardiovascular diseases [[8], [9], [10]].

Among the various TKP by-products, KYNA has been studied most. It was originally discovered in canine urine, but higher concentrations have been measured in the gut, bile, pancreatic juice of rats and pigs, and, to a lesser extent, in human saliva and synovial and amniotic fluid [11]. Its presence in many food products has also been identified. The highest concentrations are found in honeybee products, broccoli, and some potatoes [12]. Many medicinal herbs contain high KYNA concentrations, indicating therapeutic potential for the gastrointestinal system [13]. In addition to the KYNA supplied by food, it can be generated by the gut microflora.

KYNA is considered as a neuroprotector because it prevents excitotoxicity in neurons by acting as an antagonist at excitatory glutamate receptors [[14], [15], [16], [17]]. It also acts as an immunomodulator in various inflammatory diseases [18,19], and accumulating evidence suggest that KYNA may also play an important role in energy metabolism [[20], [21], [22], [23], [24], [25], [26], [27]] This last assumption is supported by several convincing arguments. TKP enzymes are expressed in tissues relevant for energy homeostasis such as adipose, skeletal muscle, liver and endocrine pancreas, blood vessel and heart [28]. Exercise shifts TKP activity toward the production of KYNA in both mouse and human muscles [26,29]. KYNA increases energy utilization by activating lipid metabolism, thermogenic, and anti-inflammatory gene expression in adipose tissue [27]. In vivo KYNA administration suppresses weight gain in rodents fed a high-fat diet and improves their glucose tolerance [27]. For these reasons, it is reasonable to ask whether KYNA can act as an anti-diabetic agent.

In the present study, we evaluated the potential antidiabetic effect of KYNA administration via drinking water in spontaneously diabetic Goto-Kakizaki (GK) rats, The adult GK rat is a well-recognized model of type 2 diabetes, and it is characterized by impaired glucose-induced insulin secretion, decreased pancreatic beta cell mass, decreased insulin sensitivity in the liver, and moderate insulin resistance in skeletal muscles and adipose tissues [30,31]. Our aim was to evaluate the effect of chronic oral KYNA administration via drinking water in GK rats: (1) on weight gain; (2) on basal plasma glucose and insulin levels; (3) on glucose tolerance; (4) on glucose-induced insulin secretion; (5) on systemic energy expenditure; (6) on gene expression signatures in liver cells.

## 2 Methods

### 2.1 Study design

In a first set of experiments aimed to evaluate the acute effect of KYNA on glucose tolerance, 20-week-old W (Wistar; Charles-River, China) and GK (Cavens, China) rats were randomly divided into two groups and fasted for 12 h. Fasting blood glucose values were measured, and each group was then respectively injected intraperitoneally with glucose (1 g/kg) + KYNA (5 mg/kg) in 0.9% NaCl, or glucose (1 g/kg) in 0.9% NaCl.

In a second set of experiments aimed to evaluate the effects of chronic KYNA administration, 6-week-old W and GK rats were randomly divided into two groups and fed with normal diet. 25 mg/L of KYNA (Sigma-Aldrich, China) or NaCl was added to the drinking water. Drinking water containing KYNA or NaCl was changed every two days. All animals were kept in a specific pathogen-free environment and fed *ad lib* and monitored during 14 weeks. Body weight, basal glycemia, and food and water intakes were measured weekly. Characteristics of the type 2 diabetic GK rat have been previously described [30,31].

In some experiments, high fat diet - streptozotocin (HFD-STZ) diabetic rat were used. To this end, 4-week-old Wistar (Charles-River, China) rats were fed on a 60% high fat diet (HFD; Trophic, China) for 8 weeks and then received 25 mg/kg *i. p.* streptozotocin (Solarbio, China). Their blood was sampled at 13 weeks of age.

In additional experiments, 12-week-old C57BL/6J male mice (Charles-River, China) were randomly assigned into two groups, in which 25 mg/L of KYNA or NaCl was added to the drinking water.

### 2.2 Glucose tolerance test

W and GK rats were each randomly divided into two groups and fasted for 12 h. Fasting blood glucose values were measured, and each group was then respectively injected intraperitoneally with glucose (1 g/kg) + KYNA (5 mg/kg) in 0.9% NaCl, or glucose (1 g/kg) in 0.9% NaCl. Blood glucose levels were measured at four time points after glucose administration (30 min, 60 min, 90 min and 120 min), using an Accu-Chek Glucometer (Accu-Chek Performa, Roche, Switzerland).

### 2.3 Systemic energy metabolism analysis

To evaluate the effects of KYNA on systemic energy metabolism, 12-week-old C57BL/6J mice were acclimated during 24 h in single cages and then monitored in a comprehensive laboratory animal monitoring system (CLAMS, Columbus Instruments, USA) for 3 days. They were randomly assigned into two groups, in which 50 mg/L of KYNA or NaCl was added to the drinking water. O_2_ consumption (VO_2_) and CO_2_ production (VCO_2_) were measured for 1 min at 14-min intervals at a flow rate of 0.72 L per minute. Respiratory Exchange Ratio (RER) was calculated by dividing VCO_2_/VO_2._ Locomotor activity, food and water intake were measured by built-in detection system. Sufficient foods and water (containing KYNA or NaCl) were provided at the beginning of monitoring. Each chamber was locked and researchers did not perform any manipulation during the whole duration of monitoring.

### 2.4 Liquid chromatograph mass spectrometry

Plasma concentrations of Trp, 5-HT, KYN and KYNA were measured by liquid chromatography-tandem mass spectrometry (LC-MS/MS). Chromatographic separation was achieved on an Atlantis dC18 reverse phase column (C18, 3 μm, 20 × 4.6 mm (int. diam.)) and elution with (A) HCO_2_H 0.1% (for positive mode on MS/MS), for 1 min and from A to (B) CH_3_CN: A (40:60, v/v) in 2 min and for 2 min in B on a Shimadzu LC20 system with a quaternary pump. MS/MS was performed on a Shimadzu LC-MS/MS 9030 system (Shimadzu, Japan). Internal standards for Trp, 5-HT, KYN and KYNA were purchased from Sigma (Sigma-Aldrich, China).

### 2.5 Cell culture

HepG2 human liver cells and AML-12 mouse liver cells (Procell, China) were cultured in MEM basal medium (Gibco, China) containing 10% fetal bovine plasma (Gibco, Australia). After reaching a certain order of magnitude in the culture flasks, the cells were inoculated into six-well plates (Corning, China) with 3 × 10^5^ cells per well, and after cell apposition, the cells were treated with KYNA for 2 and 48 h respectively. Cells were then collected to extract RNA or protein.

### 2.6 Western blotting

HepG2 cells were lysed using Cellytic MT (Sigma-Aldrich, China) and the supernatant was collected after centrifugation at 12,000 rpm. The supernatant was boiled in loading buffer (Solarbio, China) at 95 °C for 5 min. Samples were examined in SDS-PAGE. After blotting on PVDF membranes (Millipore, China), the non-specific antigen was blocked with 5% non-fat milk powder and then primary antibody anti-UCP2 (89326S, Cell Signaling Technology, America) was added overnight at 4 °C. The protein was detected using a fluorescent secondary antibody and visualized using Odyssey® system (LI-COR, USA).

### 2.7 Quantitative PCR

Total RNA was extracted from rat liver, HepG2 and AML-12 cells using the RNeasy mini Kit (Qiagen, China). cDNA for each RNA sample was synthesized using NovoScript® II Reverse Transcriptase (Novoprotein, China). The primers used for RNA were derived from human and mouse sequences, respectively, and were designed using Oligo7 software (Table S1). Real-time fluorescent quantitative qPCR reactions were performed using the QuantiNova SYBR Mixture (Qiagen, China) and Roche LightCycler® 480 system. The PCR conditions were: 95 °C for 10 min, followed by 40 cycles at 95 °C for 10 s, 60 °C for 30 s and 72 °C for 30 s mRNA transcript levels of four housekeeping genes (glyceraldehyde-3-phosphate dehydrogenase [GAPDH], TATAbox binding protein [Tbp], cyclophilin A and β-actin) were assayed.

### 2.8 RNA-sequencing procedure

Total RNA was isolated from 48 h 10 μmol/L NaCl or KYNA treated HepG2 cells using RNeasy mini kit (Qiagen, Germany). Paired-end libraries were synthesized by using the TruSeq® RNA Sample Preparation Kit (Illumina, USA) following TruSeq® RNA Sample Preparation Guide. The library was sequenced on the Illumina Novaseq6000 platform (Illumina, USA). Raw data (raw reads) in fastq format were processed using Trimmomatic, and clean reads were mapped to the human genome (GRCh38) using HISAT2 and the read counts of each gene were obtained by HTSeq-count.

The DEGs between Ctrl group and KYNA treated HepG2 were calculated by DESeq2, The Gene Ontology (GO) and Kyoto Encyclopedia of Genes and Genomes (KEGG) enrichment analyses were completed using the Metascape online analysis tools (Metascape.org).

### 2.9 Statistical analysis

All statistical analyses were performed using GraphPad Prism (GraphPad Software, California USA) and all parameters are indicated in the corresponding figure legend. Quantitative data are presented as the mean ± SEM and n is indicated for each experiment. Unpaired Student’s *t*-test was used to determine statistical significance when two groups were compared. When multiple groups are compared, a one-way ANOVA is used to determine statistical significance. Statistical significance was defined as p value < 0.05 by either test and is denoted with asterisks.

## 3 Results

### 3.1 Diabetic rats exhibit decreased plasma levels for Trp pathway metabolites including lower plasma KYNA level

**Fig. 1:**
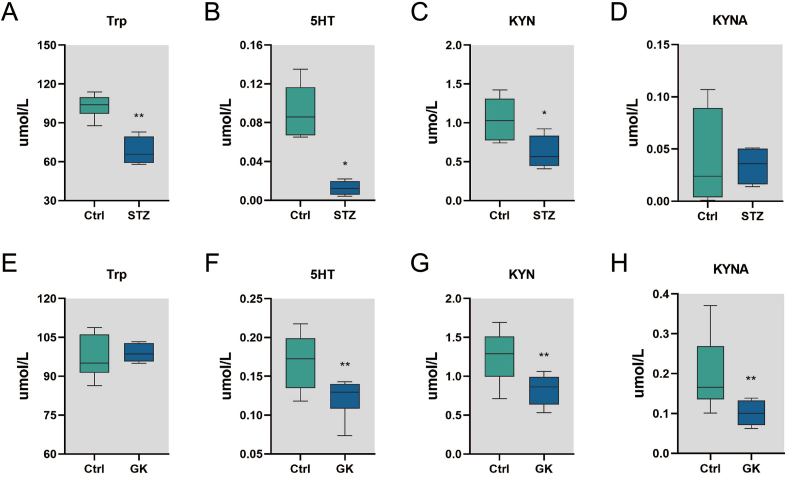
Changes in plasma Trp and its metabolites in different model of diabetes mellitus. (A–D) Comparison of Trp, 5-HT, KYN and KYNA in plasma of HFD-STZ rats (STZ) and normal W rats (Ctrl). (E–H) Comparison of Trp, 5-HT, KYN and KYNA in plasma of GK rats and normal W rats (Ctrl). Two groups were compared using the unpaired *t*-test or Mann-Whitney *U* test, as appropriate. Values are means ± SEM (n = 4–6), *p < 0.05, **p < 0.01.

We first examined the levels of plasma Trp downstream metabolites in two different rat models of type 2 diabetes, HFD-STZ rats and GK rats. As compared to levels in normal W rats, we found that the plasma levels of Trp, serotonin and KYN in the HFD-STZ model rats were significantly lower. The HFD-STZ rat also showed a lower level of KYNA but the difference did not reach statistical significance (Fig. 1A–D and Table S2). In GK rats, plasma levels of 5-HT, KYN and KYNA were significantly lower compared to those in W rats, despite normal Trp level (Fig. 1E–H and Table S3).

### 3.2 Acute administration of KYNA ameliorates glucose tolerance in diabetic GK rats

**Fig. 2:**
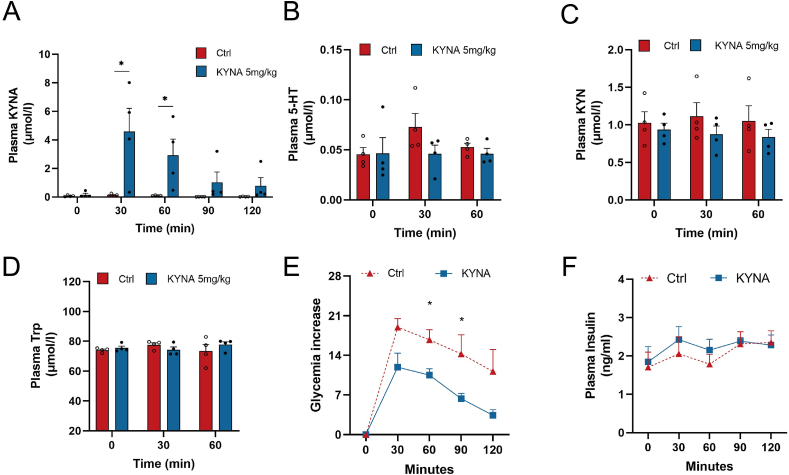
Acute administration of KYNA ameliorates glucose tolerance in diabetic GK rats. (A) Changes in plasma KYNA in GK rats over a 2-h period after intraperitoneal injection of KYNA (KYNA, 5 mg/kg) or Vehicle (Ctrl, 0.9% NaCl). (B–D) Changes in plasma 5-HT, KYN and TRP in GK rats within 1 h after intraperitoneal injection of KYNA (KYNA, 5 mg/kg) or Vehicle (Ctrl, 0.9% NaCl). (E) Blood glucose and (F) insulin changes in two groups of GK rats after intraperitoneal injection of KYNA mixed with glucose (KYNA, glucose 1 g/kg; KYNA 5 mg/kg in 0.9% NaCl) or glucose (Ctrl, Glucose 1 g/kg in 0.9% NaCl). The two groups were compared using the two-way ANOVA test. Values are means ± SEM (n = 4). *p < 0.05, **p < 0.01.

We first evaluated the acute effect of KYNA administration on glucose metabolism in diabetic GK rats. To evaluate the kinetics of an intraperitoneal KYNA dose of 5 mg/kg body weight, we quantified plasma KYNA, KYN, 5-HT and Trp up to 1 h post-injection. Explosive increase in circulating KYNA levels within 30 min of injection, while KYN, 5-HT and Trp levels remained little affected. Circulating KYNA levels increased by approximate 35 folds (0.13 v. s. 4.58 μmol/L) within 30 min after injection (Fig. 2A and Table S4), while those for KYN, 5-HT and Trp remained poorly affected (Fig. 2B–D and Table S5).

We next investigated the acute effect of KYNA on glucose tolerance and glucose-induced insulin secretion in response to i. p. co-administration of glucose (1 g/kg) and KYNA (5 mg/kg). This was done in diabetic GK rats and W rats. While KYNA treatment did not affect glucose tolerance in W rats (Fig. S1A), it improved significantly tolerance to glucose in diabetic GK rats (Fig. 2E). Since this was obtained despite any improvement of glucose-induced insulin secretion (Fig. 2F), this suggests that this anti-diabetic KYNA property targets glucose metabolism rather than insulin secretion, at least when tested under these conditions.

### 3.3 Long-term oral administration of KYNA attenuates basal hyperglycemia and delays the onset of diabetes in GK rats

**Fig. 3:**
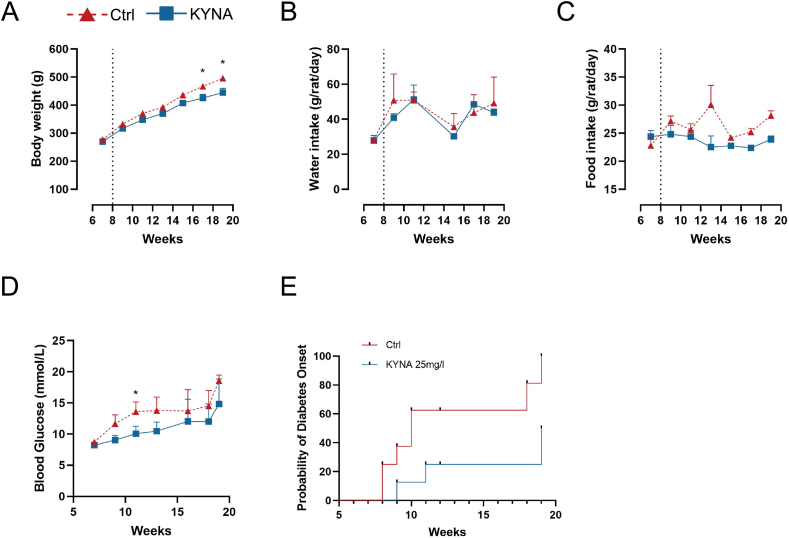
KYNA delays the onset of diabetes in GK rats. (A–C) Changes in body weight, water and food intake in two groups of GK rats after adding 25 mg/L NaCl (Ctrl) or 25 mg/L KYNA to their drinking water (KYNA) (n = 4). (D) Basal blood glucose levels on the non-fed state (i.e., at 9 a.m.) in the two groups of GK rats. (n = 4). (E) Incidence of onset of diabetes in GK rats treated by oral administration of 25 mg/L NaCl (Ctrl) or KYNA. The two groups were compared using the two-way ANOVA test. Values are means ± SEM (n = 4). *p < 0.05, **p < 0.01.

We next evaluated if chronic daily KYNA administration affects glucose metabolism in diabetic GK and normal W rats. To this end, rats were given *ad lib* access to KYNA containing drinking water (KYNA 25 mg/L) for 12 weeks. We calculated that under these conditions, the mean daily KYNA intake in GK rats amounted 1 mg/day/rat, i.e., 3 mg/kg body weight/day (a daily dosage close to that tested in the acute KYNA experiments) (Fig. 3).

In the W rats, KYNA did not affect their body weight (Fig. S1B). However, the body weight response to KYNA revealed to be different in the GK rats. While 8 weeks of KYNA administration had no significant effect on their body weight, after 12 weeks, we could observe a significant body weight reduction in KYNA-receiving GK rats when compared to NaCl-receiving GK rats (Fig. 3A). The 12 consecutive weeks of KYNA exposure did not significantly modify water intake and food intake at any time points in both W (Figs. S1C and S1D) and GK rats (Fig. 3B and C).

Basal blood glucose levels on the non-fed state (i.e., at 9 a.m.) in the GK rats were also monitored sequentially to determine the effect of chronic KYNA on the development of diabetes in the GK rats. We found that KYNA significantly decreased basal blood glucose after 2 weeks of treatment (10 weeks old) (Fig. 3D). We then recorded the disease onset time of GK rats. In this study, onset of diabetes was considered to be established when three consecutive independent blood glucose measurements were greater than 11.1 mmol/L. We found that some GK rats began to develop diabetes from the age of 8 weeks, and by the age of 19 weeks, all GK rats in control group had developed diabetes (Ctrl group). By contrast, only 50% of the KYNA group had developed diabetes by the age of 19 weeks. Kaplan-Meier survival test showed that KYNA treatment significantly delayed the onset of diabetes in GK rats (Fig. 3E).

Furthermore, such KYNA effect is diabetes-related since in W rats, no significant change of blood glucose could be detected when comparing the KYNA-receiving W rats to those receiving NaCl. In other words, long-term oral KYNA was able to delay the onset of diabetes in GK rats, while it had no effect in normal W rats.

### 3.4 KYNA administration increases respiratory exchange ratio

**Fig. 4:**
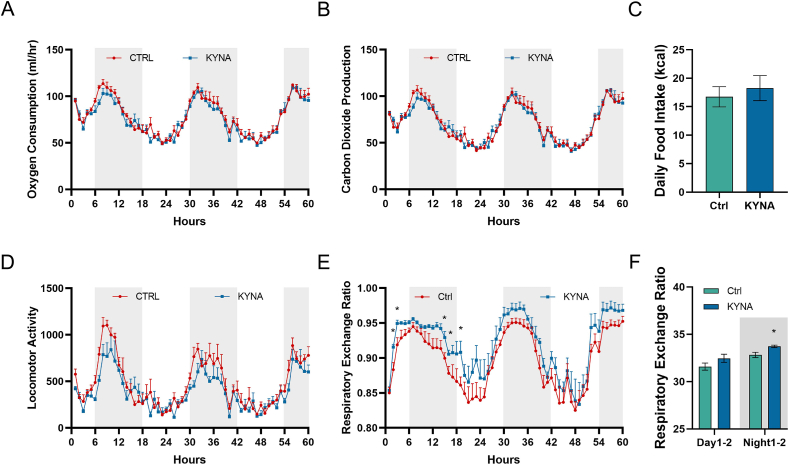
Effect of KYNA on the overall metabolism of C57BL/6J mice. 25 mg/L KYNA or NaCl (Ctrl) were added to the drinking water of C57BL/6J mice and placed in separate CLAMS metabolic cages. (A) Oxygen consumption. (B) Carbon dioxide production. (C) Food intake. (D) Locomotor activity. (E) Respiratory exchange ratios. (F) Comparison of respiratory exchange rates between experimental and control groups for the first 2 days of the experiment. White and gray zone indicates respectively the day and night. Statistical analysis using *t*-test (n = 5), *p < 0.05.

To further assess the effect of KYNA on systemic energy metabolism, we examined the energy expenditure in C57BL/6J mice, using indirect calorimetry. Mice were randomly assigned into two groups and 25 mg/L KYNA or NaCl was added to the drinking water respectively. Additionally, mice were fasted overnight in the metabolic cage to ensure that mice start eating and drinking from the same moment. The data were recorded for 3 consecutive days from the time the mice started to eat and drink. We did not find any difference in VO_2_, VCO_2_ between the two groups during whole experiment (Fig. 4A and B). Also, we did not observe significant changes in locomotion, food and water intakes (Fig. 4C and D). However, when we calculate the respiratory exchange ratio (VCO_2_/VO_2_), we found its value increased significantly in the KYNA group, especially during the dark period (Fig. 4E and F). A higher RER indicates that the body is burning more carbohydrates while lower RER indicates that the body is burning more lipid due to the difference of oxygen content between the two types of fuels. Given the fact that mice are more active during the dark period, these observations suggest that KYNA could promote glucose utilization and energy metabolism during active phase.

### 3.5 KYNA enhances mRNA and protein expression of hepatic uncoupling protein genes

With a view to understand the mechanisms by which KYNA affects energy metabolism, we started our investigations by focusing on the liver, as it is the main site of tryptophan and kynurenines metabolism, the hub of energy metabolism, and a major player in the development of diabetes.

We have evaluated the direct effects of KYNA on liver cells *in vitro.* Human HepG2 cells and mouse AML-12 cells were treated for 2 h or 48 h with different concentrations of KYNA (10 and 100 μmol/L). We then examined expression of gene related to energy metabolism, oxidative stress, inflammatory response and tryptophan metabolism.

**Fig. 5:**
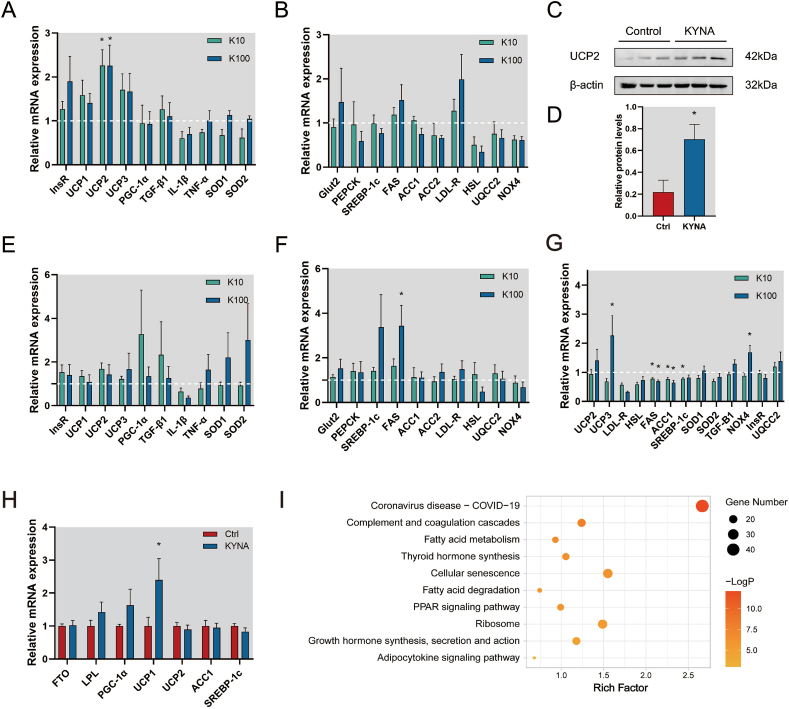
KYNA enhances mRNA and protein expression of hepatic UCP genes. (A, B) Real-time quantitative PCR analysis (n = 6) of gene expression in HepG2 cells after 48 h of 10 μmol/L (K10) or 100 μmol/L (K100) KYNA treatment compared with control group (white punctuated line). (C, D) Western blot analysis of UCP2 expression in HepG2 cells after 48 h of 100 μmol/L KYNA treatment (n = 3), (E, F) Real-time quantitative PCR analysis of gene expression in HepG2 cells after 2 h treatment with 10 μmol/L (K10) or 100 μmol/L (K100) KYNA (n = 6), (G) Quantitative PCR analysis of gene expression in AML-12 cells after 48 h treatment with 10 μmol/L (K10) or 100 μmol/L (K100) KYNA (n = 6), (H) Expression of hepatic energy metabolism genes in GK rats after oral administration 25 mg/kg KYNA (KYNA) or NaCl (Ctrl) (I) Comprehensive analysis of transcriptomic pathways in KYNA-treated HepG2 cells after 48 h KYNA, 100 μmol/L (n = 3). Statistical analysis using *t*-test, *p < 0.05.

In HepG2 cells, a 48 h treatment of cells with KYNA induced a unique increase of UCP1, UCP2 and UCP3 expressions, with the UCP2 mRNA increase reaching statistical significance (Fig. 5A and B). We found similar results at the protein level, with significantly higher level of UCP2 in KYNA-exposed HepG2 cells (Fig. 5C and D). However, 2 h short treatment had no such effect (Fig. 5E and F). A similar pattern was observed in the AML-12 cells exposed to KYNA for 48 h and in the livers of GK rats treated with KYNA for 4 weeks. In the case of AML-12 cells, instead of UCP2, we found that UCP3 expression was significantly increased after 48 h of KYNA treatment (Fig. 5G). In the GK rat livers, we observed a significant increase in UCP1 expression following oral administration of KYNA (Fig. 5H).

To further investigate the impact of KYNA on liver, we conducted a transcriptomic analysis of HepG2 cells following 48 h of KYNA exposure. We found 130 downregulated genes and 145 upregulated genes in KYNA-treated cells compared to untreated cells. The top 10 enriched pathways are presented in Fig. 5I, among which, we noticed that the fatty acid metabolism and PPAR signaling pathways were highly enriched, these two pathways bing closely associated to thermogenesis and UCPs expression. These observations suggest KYNA enhances hepatic energy metabolism through promoting UCP gene expression.

Overall, our observations suggest that hyperglycemic rodents showed a decreased plasma KYNA levels and oral administration of KYNA significantly delays diabetes onset in pre-diabetic GK rats. We further demonstrated that oral KYNA administration significant increased respiration exchange ratio. This effect could be related to the enhancement of UCPs expression and energy metabolism-related gene expression in the liver.

## 4 Discussion

Obesity and diabetes are two of the most pressing health concerns facing populations around the world. In the present study, we found that hyperglycemic GK rats had lower plasmatic KYNA levels, and that long-term oral KYNA administration in pre-diabetic GK rat significantly delayed diabetes onset. In further studies, we also demonstrated that KYNA may induce a sustained increase in respiratory exchange rate, which may be due to KYNA stimulating hepatic UCPs and energy metabolism. Overall, these findings suggest that KYNA could serve as an anti-diabetic agent.

In our study, we noted a decrease in plasma KYNA levels in hyperglycemic rat models. However, there is controversy surrounding the plasma levels of KYNA in clinical settings. Studies have indicated that KYNA is positively correlated with BMI, potentially due to its increased biosynthesis in omental adipose tissue [32,33]. In contrast, Pyun et al. found a negative correlation between serum KYNA levels and BMI [34]. These controversial observations may be related to differences in the inclusion criteria. Moreover, as KYNA can be rapidly eliminated through urine (with a half-life of approximately 30 min according to our i.p. KYNA data), the decreased KYNA levels in our hyperglycemic animal model may be attributed to hyperglycemia-associated polyuria.

We found that the addition of KYNA to drinking water slightly inhibited weight gain in diabetes GK rats. Similarly, a recent study, in which KYNA was added to the drinking water of Wistar rats three weeks after birth, found that weight gain was reduced without affecting skeletal development [35,36]. However, in our study, we did not find any significant effect in healthy Wistar rats. This may be due to the fact that the time we started the intervention was later than the above-mentioned study. At 7 weeks of age, the rats' growth rate had already slowed down. Another study, in which KYNA was administered intraperitoneally to C57BL/6J mice for 3 weeks, found a difference in body weight after two weeks compared to controls [27]. This may be due to the higher KYNA bioavailability and higher plasma peak levels by intraperitoneal injections. Nevertheless, these studies suggest that KYNA plays a role in body weight restriction, whether given orally or intraperitoneally.

Next, we found that the addition of KYNA to drinking water significantly delay the onset of diabetes in GK rats. Although it has been reported that intraperitoneal administration of KYNA can improve glucose tolerance in HFD-fed overweight mice [27], we have demonstrated for the first time that this compound can delay the onset of diabetes using a diabetic model. We further found that the impaired glucose tolerance in the KYNA group was improved in the glucose tolerance of GK rats during the IPGTT assay, which supports the role of KYNA in type 2 diabetes.

Since fats and carbohydrates differ in their oxygen content, the respiratory quotient predicts whether these two substances will be used as fuel [[37], [38], [39]]. In our study of mice receiving KYNA in their drinking water, the respiratory quotient increased at night and was significantly different from the control group, while activity and food intake were not affected. These data suggest that KYNA may stimulate glucose oxidation in mice after meals and during activity. Accordingly, we found an upregulation of UCPs which may play an important role in delaying the onset of diabetes and maintaining glucose homeostasis.

KYNA is a potent agonist of the GPR35 receptor [40]. The GPR35 receptor is considered a potential target for the treatment of diabetes, hypertension and asthma [27,41]. It can be hypothesized that KYNA maintained glucose homeostasis in the glucose tolerance assay by activating the GPR35 receptor. However, to further confirm this hypothesis, studies with GPR35 knockout mice are needed.

Agudelo et al. found that KYNA can activate the GPR35 receptor in adipocytes, increase UCP expression and induce adipose tissue browning [27]. In the present study, we found KYNA can also induce an increase in UCP expression in hepatocytes. In HepG2 cells, KYNA significantly enhanced UCP2 expression, whereas it was UCP3 for AML-12 cell and UCP1 for GK rats, this may be due to the difference in species. Mitochondria store energy in their inner membrane in the form of proton gradients, and available evidence suggests that all UCPs can dissipate gradients to generate heat or regulate metabolite fluxes. Also, UCPs prevents fatty acid anion accumulation, reduces damage to mitochondria improve insulin resistance [42,43].

### 4.1 Limitation of study

This study also has several limitations. Firstly, we did not measure changes in urinary output in the experimental animals. Given that KYNA may lead to natriuresis [44], our study data are not sufficient to fully understand the role of KYNA. Secondly, our study was limited to the liver, and the effects of KYNA in other organs in the body require further investigation. Lastly, this study is limited to the GK model to investigate the role of KYNA in delaying the onset of diabetes, and future research needs to test other diabetic models.

## 5 Conclusion

Overall, the findings of this study suggest that oral administration of KYNA significantly increases the respiratory exchange rate and delays diabetes onset in a rodent model of type 2 diabetes. This effect is obtained in the presence of enhanced UCPs expression and expression of energy metabolism-related gene in the liver. Since KYNA is a naturally endogenous metabolite of tryptophan and a small molecule that can be absorbed orally, it has outstanding advantages in terms of translation potential compared to other anti-diabetic compounds. We propose that KYNA may have promising potential as an anti-diabetic agent and warrants further investigation with the aim to develop of novel therapeutic strategy.

## Ethics statement

All procedures for care and use of animals were approved by the Shandong Institute of Endocrine and Metabolic Diseases Laboratory Animal Ethics Committee (Approval No.20200501) and all applicable institutional and governmental regulations concerning the ethical use of animals were followed.

## Author agreement statement

We (Delong Zhen, Lina Ding, Bao Wang, Xiaolei Wang, Yanli Hou, Wenyu Ding, Bernard Portha and Junjun Liu) the undersigned declare that this manuscript is original, has not been published before and is not currently being considered for publication elsewhere. We confirm that the manuscript has been read and approved by all named authors and that there are no other persons who satisfied the criteria for authorship but are not listed. We further confirm that the order of authors listed in the manuscript has been approved by all of us.

## Author contributions

Z.D. and L.D.: methodology, formal analysis, writing – original draft. B.W. and L.D.: resources, data curation. X.W. and Y.H.: investigation, validation. W.D.: resources, supervision. B.P.: conceptualization, writing, review, editing. J.L.: conceptualization, funding, writing, review, editing, supervision.

## Declaration of competing interest

The authors declare that they have no known competing financial interests or personal relationships that could have appeared to influence the work reported in this paper

## References

[1] Ostapiuk A., Urbanska E.M. (2022). Kynurenic acid in neurodegenerative disorders—unique neuroprotection or double-edged sword?. CNS Neurosci. Ther..

[2] Savitz J. (2020). The kynurenine pathway: a finger in every pie. Mol. Psychiatr..

[3] Badawy A.A.B. (2017). Kynurenine pathway of tryptophan metabolism: regulatory and functional aspects. Int. J. Tryptophan Res..

[4] Modoux M., Rolhion N., Mani S., Sokol H. (2021). Tryptophan metabolism as a pharmacological target. Trends Pharmacol. Sci..

[5] Lahiri P., Dhaware D., Singh A., Panchagnula V., Ghosh D. (2019).

[6] Galderisi A., Pirillo P., Moret V., Stocchero M., Gucciardi A., Perilongo G., Moretti C., Monciotti C., Giordano G., Baraldi E. (2018). Metabolomics reveals new metabolic perturbations in children with type 1 diabetes. Pediatr. Diabetes.

[7] Oxenkrug G.F. (2015). Increased plasma levels of xanthurenic and kynurenic acids in type 2 diabetes. Mol. Neurobiol..

[8] Cason C.A., Dolan K.T., Sharma G., Tao M., Kulkarni R., Helenowski I.B., Doane B.M., Avram M.J., McDermott M.M., Chang E.B., Ozaki C.K., Ho K.J. (2018). Plasma microbiome-modulated indole- and phenyl-derived metabolites associate with advanced atherosclerosis and postoperative outcomes. J. Vasc. Surg..

[9] Nagy B.M., Nagaraj C., Meinitzer A., Sharma N., Papp R., Foris V., Ghanim B., Kwapiszewska G., Kovacs G., Klepetko W., Pieber T.R., Mangge H., Olschewski H., Olschewski A. (2017). Importance of kynurenine in pulmonary hypertension. Am. J. Physiol. Lung Cell Mol. Physiol..

[10] Razquin C., Ruiz-Canela M., Toledo E., Hernández-Alonso P., Clish C.B., Guasch-Ferré M., Li J., Wittenbecher C., Dennis C., Alonso-Gómez A., Fitó M., Liang L., Corella D., Gómez-Gracia E., Estruch R., Fiol M., Lapetra J., Serra-Majem L., Ros E., Aros F., Salas-Salvadó J., Hu F.B., Martínez-González M.A. (2021). Metabolomics of the tryptophan–kynurenine degradation pathway and risk of atrial fibrillation and heart failure: potential modification effect of Mediterranean diet. Am. J. Clin. Nutr..

[11] Turski M.P., Turska M., Paluszkiewicz P., Parada-Turska J., Oxenkrug G.F. (2013). Kynurenic acid in the digestive system–-new facts, new challenges. Int. J. Tryptophan Res..

[12] Turski M.P., Turska M., Zgrajka W., Kuc D., Turski W.A. (2009). Presence of kynurenic acid in food and honeybee products. Amino Acids.

[13] Turski M.P., Turska M., Zgrajka W., Bartnik M., Kocki T., Turski W.A. (2011). Distribution, synthesis, and absorption of kynurenic acid in plants. Planta Med..

[14] Schwarcz R., Stone T.W. (2017). The kynurenine pathway and the brain: challenges, controversies and promises. Neuropharmacology.

[15] Cervenka I., Agudelo L.Z., Ruas J.L. (2017). Kynurenines: tryptophan’s metabolites in exercise, inflammation, and mental health. Science (1979).

[16] Gigler G., Szénási G., Simó A., Lévay G., Hársing L.G., Sas K., Vécsei L., Toldi J. (2007). Neuroprotective effect of L-kynurenine sulfate administered before focal cerebral ischemia in mice and global cerebral ischemia in gerbils. Eur. J. Pharmacol..

[17] Luchowska E., Luchowski P., Sarnowska A., Wielosz M., Turski W.A., Urbañska E.M. (2003). Short communication endogenous level of kynurenic acid and activities of kynurenine aminotransferases following transient global ISCHEMIA in the GERBIL HIPPOCAMPUS. Pol. J. Pharmacol..

[18] Wirthgen E., Hoeflich A., Rebl A., Günther J. (2018). Kynurenic Acid: the Janus-faced role of an immunomodulatory tryptophan metabolite and its link to pathological conditions. Front. Immunol..

[19] Favennec M., Hennart B., Caiazzo R., Leloire A., Yengo L., Verbanck M., Arredouani A., Marre M., Pigeyre M., Bessede A., Guillemin G.J., Chinetti G., Staels B., Pattou F., Balkau B., Allorge D., Froguel P., Poulain-Godefroy O. (2015). The kynurenine pathway is activated in human obesity and shifted toward kynurenine monooxygenase activation. Obesity.

[20] Matsuoka K., Kato K., Takao T., Ogawa M., Ishii Y., Shimizu F., Masuda J., Takada A. (2017). Concentrations of various tryptophan metabolites are higher in patients with diabetes mellitus than in healthy aged male adults. Diabetol. Int..

[21] Favennec M., Hennart B., Caiazzo R., Leloire A., Yengo L., Verbanck M., Arredouani A., Marre M., Pigeyre M., Bessede A., Guillemin G.J., Chinetti G., Staels B., Pattou F., Balkau B., Allorge D., Froguel P., Poulain-Godefroy O. (2015). The kynurenine pathway is activated in human obesity and shifted toward kynurenine monooxygenase activation. Obesity.

[22] Oxenkrug G.F. (2015). Increased plasma levels of xanthurenic and kynurenic acids in type 2 diabetes. Mol. Neurobiol..

[23] Yu E., Papandreou C., Ruiz-Canela M., Guasch-Ferre M., Clish C.B., Dennis C., Liang L., Corella D., Fitó M., Razquin C., Lapetra J., Estruch R., Ros E., Cofán M., Arós F., Toledo E., Serra-Majem L., Sorlí J.v., Hu F.B., Martinez-Gonzalez M.A., Salas-Salvado J. (2018). Association of tryptophan metabolites with incident type 2 diabetes in the PREDIMED trial: a case–cohort study. Clin. Chem..

[24] Eng J.M., Estall J.L. (2021). Diet-induced models of non-alcoholic fatty liver disease: food for thought on sugar, fat, and cholesterol. Cells.

[25] Manka P., Syn W.K. (2021). NASH, metabolic syndrome, and diabetes: how sugar and fat increase the risk of developing advanced liver disease. Dig. Dis. Sci..

[26] Agudelo L.Z., Femenía T., Orhan F., Porsmyr-Palmertz M., Goiny M., Martinez-Redondo V., Correia J.C., Izadi M., Bhat M., Schuppe-Koistinen I., Pettersson A.T., Ferreira D.M.S., Krook A., Barres R., Zierath J.R., Erhardt S., Lindskog M., Ruas J.L. (2014). Skeletal muscle PGC-1α1 modulates kynurenine metabolism and mediates resilience to stress-induced depression. Cell.

[27] Agudelo L.Z., Ferreira D.M.S., Cervenka I., Bryzgalova G., Dadvar S., Jannig P.R., Pettersson-Klein A.T., Lakshmikanth T., Sustarsic E.G., Porsmyr-Palmertz M., Correia J.C., Izadi M., Martínez-Redondo V., Ueland P.M., Midttun Ø., Gerhart-Hines Z., Brodin P., Pereira T., Berggren P.O., Ruas J.L. (2018). Kynurenic acid and Gpr35 regulate adipose tissue energy homeostasis and inflammation. Cell Metabol..

[28] Liu J.J., Movassat J., Portha B. (2019). Emerging role for kynurenines in metabolic pathologies. Curr. Opin. Clin. Nutr. Metab. Care.

[29] Schlittler M., Goiny M., Agudelo L.Z., Venckunas T., Brazaitis M., Skurvydas A., Kamandulis S., Ruas J.L., Erhardt S., Westerblad H., Andersson D.C. (2016). Endurance exercise increases skeletal muscle kynurenine aminotransferases and plasma kynurenic acid in humans. Am. J. Physiol. Cell Physiol..

[30] Portha B., Lacraz G., Kergoat M., Homo-Delarche F., Giroix M.H., Bailbé D., Gangnerau M.N., Dolz M., Tourrel-Cuzin C., Movassat J. (2009). The GK rat beta-cell: a prototype for the diseased human beta-cell in type 2 diabetes?. Mol. Cell. Endocrinol..

[31] Portha B., Giroix M.-H., Tourrel-Cuzin C., Le-Stunff H., Movassat J. (2012).

[32] Favennec M., Hennart B., Caiazzo R., Leloire A., Yengo L., Verbanck M., Arredouani A., Marre M., Pigeyre M., Bessede A., Guillemin G.J., Chinetti G., Staels B., Pattou F., Balkau B., Allorge D., Froguel P., Poulain-Godefroy O. (2015). The kynurenine pathway is activated in human obesity and shifted toward kynurenine monooxygenase activation. Obesity.

[33] Ho J.E., Larson M.G., Ghorbani A., Cheng S., Chen M.H., Keyes M., Rhee E.P., Clish C.B., Vasan R.S., Gerszten R.E., Wang T.J. (2016). Metabolomic profiles of body mass index in the framingham heart study reveal distinct cardiometabolic phenotypes. PLoS One.

[34] Pyun D.H., Kim T.J., Kim M.J., Hong S.A., Abd El-Aty A.M., Jeong J.H., Jung T.W. (2021). Endogenous metabolite, kynurenic acid, attenuates nonalcoholic fatty liver disease via AMPK/autophagy- and AMPK/ORP150-mediated signaling. J. Cell. Physiol..

[35] Tomaszewska E., Muszyński S., Kuc D., Dobrowolski P., Lamorski K., Smolińska K., Donaldson J., Świetlicka I., Mielnik-Błaszczak M., Paluszkiewicz P., Parada-Turska J. (2019). Chronic dietary supplementation with kynurenic acid, a neuroactive metabolite of tryptophan, decreased body weight without negative influence on densitometry and mandibular bone biomechanical endurance in young rats. PLoS One.

[36] Milart P., Paluszkiewicz P., Dobrowolski P., Tomaszewska E., Smolinska K., Debinska I., Gawel K., Walczak K., Bednarski J., Turska M., Raban M., Kocki T., Turski W.A. (2019). Kynurenic acid as the neglected ingredient of commercial baby formulas. Sci. Rep..

[37] Charrière N., Montani J.P., Dulloo A.G. (2016). Postprandial thermogenesis and respiratory quotient in response to galactose: Comparison with glucose and fructose in healthy young adults. J. Nutr. Sci..

[38] Goldenshluger A., Constantini K., Goldstein N., Shelef I., Schwarzfuchs D., Zelicha H., Meir A.Y., Tsaban G., Chassidim Y., Gepner Y. (2021). Effect of dietary strategies on respiratory quotient and its association with clinical parameters and organ fat loss: a randomized controlled trial. Nutrients.

[39] Macdonald I. (1984). Differences in dietary-induced thermogenesis following the ingestion of various carbohydrates. Ann. Nutr. Metab..

[40] Milligan G. (2011). Orthologue selectivity and ligand bias: translating the pharmacology of GPR35. Trends Pharmacol. Sci..

[41] Divorty N., Mackenzie A.E., Nicklin S.A., Milligan G. (2015). G protein-coupled receptor 35: an emerging target in inflammatory and cardiovascular disease. Front. Pharmacol..

[42] Schrauwen P., Hesselink M.K.C. (2003). The role of uncoupling protein 3 in fatty acid metabolism: protection against lipotoxicity?. Proc. Nutr. Soc..

[43] Schrauwen P., Hesselink M.K.C., Vaartjes I., Kornips E., Saris W.H.M., Giacobino J.-P., Russell A. (2002). Effect of acute exercise on uncoupling protein 3 is a fat metabolism-mediated effect. Am. J. Physiol. Endocrinol. Metabol..

[44] Bądzyńska B., Zakrocka I., Sadowski J., Turski W.A., Kompanowska-Jezierska E. (2014). Effects of systemic administration of kynurenic acid and glycine on renal haemodynamics and excretion in normotensive and spontaneously hypertensive rats. Eur. J. Pharmacol..

