# Supplementary material for: Oral administration of kynurenic acid delays the onset of type 2 diabetes in Goto-Kakizaki rats
Source: Heliyon. 2023 Jun 27;9(7):e17733. doi: 10.1016/j.heliyon.2023.e17733 (PMC10328841; doi:10.1016/j.heliyon.2023.e17733)
Supplement: Multimedia component 1 [file mmc1.docx]

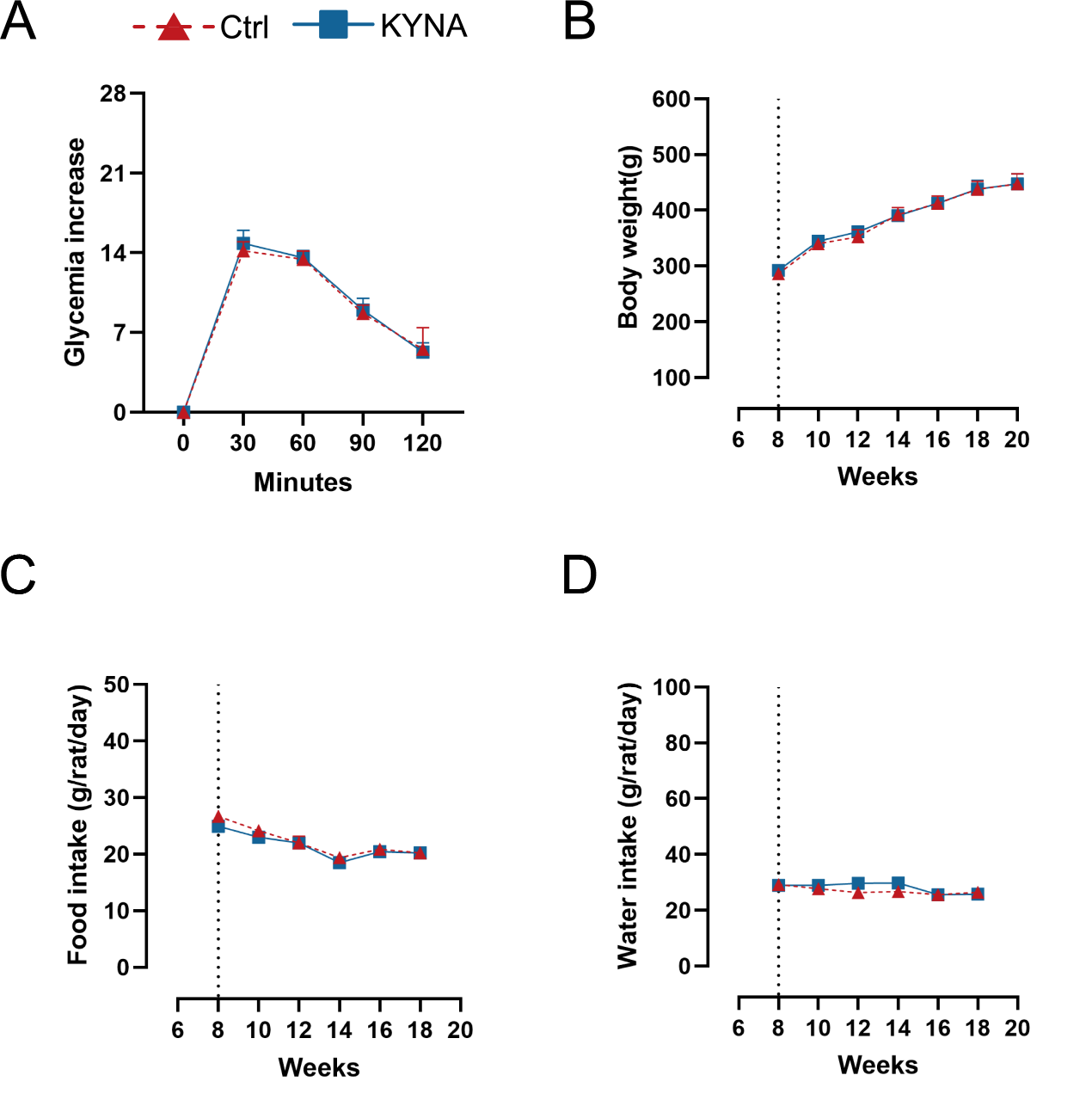


**Figure S1. Effects of KYNA on Wistar rats in the short and long term**

(A) Blood glucose changes in two groups of Wistar rats after intraperitoneal injection of glucose (glucose 1 g/Kg in 0.9% NaCl) or KYNA mixed with glucose (glucose 1 g/Kg; KYNA 5 mg/Kg in 0.9% NaCl) (n=5). (B-D) Changes in body weight, food and water intake in two groups of Wistar rats after adding 25mg/L NaCl and KYNA to their drinking water (n=5).

**Table S1. Transcripts analyzed by real-time PCR, gene symbols and primer sequences.**

| Target transcript | Polarity | Sequence (5′→3′) |
| --- | --- | --- |
| UCP1 | Sense | GCCTCTACGACACGGTCCA |
|  | Anti-sense | GTCCCTTTCCAAAGACCCGTCA |
| UCP2 | Sense | CCTATTCCGGCAGAGTTCCT |
|  | Anti-sense | GCTGATTTCCTGCTACGTCCC |
| UCP3 | Sense | GCCAGATGAGCTTCGCCTCC |
|  | Anti-sense | CGTCCATAGTCCCGCTGT |
| LDL-R | Sense | ACCTGTCGCCCTGACGAA |
|  | Anti-sense | CCAACTTCATCGCTCATGTCCT |
| Glut2 | Sense | ATAGCCATCTTCCTCTTTGTCAG |
|  | Anti-sense | TAAGGTCCACAGAAGTCCGCAA |
| GCK | Sense | AAACCCCAAACCAGCCCGAG |
|  | Anti-sense | CTCCATCCTGGCTCTGTCGTC |
| Ins-R | Sense | TGTTCCCCAACCTCACGGTC |
|  | Anti-sense | ATGTCTCCACACTCCTCGTT |
| PEPCK1 | Sense | ACGCCACCATAATAATCATCACC |
|  | Anti-sense | AAAGATCAATGCCTGAGTAACCT |
| SOD1 | Sense | TGCAGGTCCTCACTTTAATCCTC |
|  | Anti-sense | ATTTCCACCTTTGCCCAAGTCA |
| SOD2 | Sense | CCCGACCTGCCCTACGACT |
|  | Anti-sense | CTCCACCACCGTTAGGGCTG |
| SREBP-1C | Sense | GGAGGGGTAGGGCCAACGGCCT |
|  | Anti-sense | CATGTCTTCGAAAGTGCAATCC |
| FAS | Sense | CGGAAACTGCAGGAGCTGTC |
|  | Anti-sense | CACGGAGTTGAGCCGCAT |
| ACC1 | Sense | GAATGTTTGGGGATATTTCAG |
|  | Anti-sense | TTCTGCTATCAGTCTGTCCAG |
| ACC2 | Sense | CGCATTTACCGTCACTTGGAAC |
|  | Anti-sense | GTCCGTCACTTCCACACCT |
| TGF-β1 | Sense | CTAATGGTGGAAACCCACAACG |
|  | Anti-sense | TATCGCCAGGAATTGTTGCTG |
| PGC1-α | Sense | CATAAAGCCAACCAAGATAACCC |
|  | Anti-sense | GGTCTTCCTTTCCTCGTGTCCA |
| UQCC2 | Sense | GCCTCTATCCCTGACCTTGC |
|  | Anti-sense | CTGTCCCTCACGGTTCCAG |
| NOX4 | Sense | CCGAACACTCTTGGCTTACCTCC |
|  | Anti-sense | CAGCCACATGCACGCCTGA |
| IL-1β | Sense | CCCTCTGTCATTCGCTCCC |
|  | Anti-sense | TAAAGAGAGCACACCAGTCCA |
| TNF-α | Sense | GAACCCCGAGTGACAAGCCT |
|  | Anti-sense | TATCTCTCAGCTCCACGCCAT |
| HSL | Sense | CCTCAAGGCTCATCCACAACA |
|  | Anti-sense | TTGTCCTCCGCCAGAGTCAC |

**Table S2 Trp and its downstream metabolites in plasma of Wistar and HFD-STZ rats related to figure 1A**

|  | Trp(μmol/L) | 5-HT(μmol/L) | KYN(μmol/L) | KYNA(μmol/L) |
| --- | --- | --- | --- | --- |
| Wistar | 68.077±4.731 | 0.013±0.003 | 0.616±0.094 | 0.039±0.021 |
| HFD-STZ | 102.838±3.939 | 0.091±0.011 | 1.04±0.112 | 0.034±0.008 |
| P value | <0.0001 | 0.0011 | 0.0427 | 0.8587 |

**Table S3 Trp and its downstream metabolites in plasma of Wistar and GK rats related to figure 1B**

|  | Trp (μmol/L) | 5-HT(μmol/L) | KYN(μmol/L) | KYNA(μmol/L) |
| --- | --- | --- | --- | --- |
| Wistar | 97.423±2.183 | 0.169±0.01 | 1.225±0.09 | 0.199±0.024 |
| GK | 99.032±1.18 | 0.123±0.008 | 0.823±0.062 | 0.101±0.01 |
| P value | 0.5454 | 0.0045 | 0.0057 | 0.0026 |

**Table S4 Changes in plasma KYNA (μmol/L) in GK rats over a 2-hour period after intraperitoneal injection of KYNA related to figure 2**

| Time (min) | 0 | 30 | 60 | 90 | 120 |
| --- | --- | --- | --- | --- | --- |
| Ctrl | 0.07±0.019 | 0.13±0.036 | 0.088±0.009 | 0.019±0.002 | 0.027±0.005 |
| KYNA | 0.155±0.085 | 4.582±1.412 | 2.909±0.994 | 1.025±0.628 | 0.79±0.493 |
| P value | 0.425812 | 0.034212 | 0.049265 | 0.214956 | 0.22864 |

**Table S5 Changes in plasma Trp metabolites in GK rats within 1 h after intraperitoneal injection of KYNA related to figure 2**

|  | Metabolite |  | **Time (min)** |  |
| --- | --- | --- | --- | --- |
|  |  | 0 | 30 | 60 |
|  | KYN (μmol/L) | 1.028±0.127 | 1.114±0.158 | 1.05±0.177 |
| Ctrl | 5-HT (μmol/L) | 0.046±0.006 | 0.073±0.012 | 0.053±0.003 |
|  | Trp (μmol/L) | 74.065±0.604 | 77.626±1.177 | 73.428±3.866 |
|  | KYN (μmol/L) | 0.936±0.075 | 0.873±0.096 | 0.838±0.087 |
| KYNA | 5-HT (μmol/L) | 0.047±0.014 | 0.046±0.008 | 0.046±0.005 |
|  | Trp (μmol/L) | 75.526±1.128 | 74.332±1.64 | 77.711±1.629 |
|  | KYN | 0.608161 | 0.301378 | 0.386701 |
| P value | 5-HT | 0.955119 | 0.148778 | 0.342463 |
|  | Trp | 0.360523 | 0.207404 | 0.410709 |
